# Aging‐related biomarkers in testicular cancer survivors after different oncologic treatments

**DOI:** 10.1002/cam4.70200

**Published:** 2024-09-20

**Authors:** A. Carballo‐Muñoz, G. Lima, L. Llorente, Y. A. Remolina‐Bonilla, S. Jaime‐Casas, A. Otamendi‐Lopez, R. A. Ortiz‐Guerra, Hugo E. Velazquez, Y. Atisha‐Fregoso, M. T. Bourlon

**Affiliations:** ^1^ Department of Hematology and Oncology Instituto Nacional de Ciencias Médicas y Nutrición Salvador Zubirán Mexico City Tlalpan Mexico; ^2^ Department of Inmunology and Rheumatology Instituto Nacional de Ciencias Médicas y Nutrición Salvador Zubirán Mexico City Tlalpan Mexico; ^3^ Radiology Department National Institute of Cardiology Mexico City Tlalpan Mexico; ^4^ Institute of Molecular Medicine, Feinstein Institutes for Medical Research New York New York USA

**Keywords:** biomarkers, cancer care continuum, chemotherapy, germ cell tumor, survival

## Abstract

**Purpose:**

Testicular cancer survivors (TCS) exposed to chemotherapy have an increased expression of CDKN2A/*p16* ^*INK4a*^ and a lymphocyte phenotype associated with immunosenescence. We seek to define whether the immunosenescent phenotype is associated with chemotherapy.

**Methods:**

Case–control study of TCS, disease‐free ≥3 months and stratified by primary treatment modality into orchiectomy only, chemotherapy, or bone marrow transplant (BMT). Each group was compared with age‐matched healthy controls (HC). We measured the relative proportions of lymphocyte subpopulations using flow cytometry, levels of C‐reactive protein, and relative expression of *CDKN2A/p16* ^*INK4a*^ quantified by qPCR.

**Results:**

We included 65 patients; 19 were treated with orchiectomy only, 35 received different doses of chemotherapy, and 11 underwent BMT. The chemotherapy and BMT groups had decreased naïve CD4 cells compared to HC. The chemotherapy group showed increased central and effector memory CD4 cells, as well as effector and terminally differentiated CD8 cells, compared to HC. Chemotherapy (chemotherapy 1.84 vs. HC 0.92; *p* < 0.01) and BMT (BMT 6.96 vs. HC 1.25; *p* < 0.005) groups had higher expression of *CDKN2A*/*p16* ^*INK4a*^ compared to HC. The orchiectomy group showed no significant difference with HC (orchiectomy 1.73 vs. HC 1.01; *p* = 0.17). CRP levels were higher in all groups when compared with HC; in the orchiectomy group, they were only marginally increased (chemotherapy 0.22 vs. HC 0.06; *p* < 0.01; BMT 0.26 vs. HC 0.06; *p* < 0.01; orchiectomy 0.09 vs. HC 0.07; *p* < 0.01).

**Conclusions:**

Among TCS, only patients exposed to cytotoxic agents developed an immunosenescent phenotype. This finding supports the attribution of this alteration to the cytotoxic treatment.

## Graphical abstract

There are alterations in the immune system of testicular cancer survivors that resembles to what happens with aging, it was not known if these changes are caused by treatment. In this report, we looked at the effect that different treatment modalities have on testicular cancer survivors, and we found that only patients exposed to chemotherapy have markers of cellular aging.

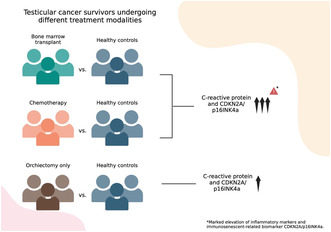

## 1 INTRODUCTION

Germ cell testicular cancer (TC) is the most common solid malignancy affecting males between the ages of 15 and 35 years.^1^ Therapeutic advancements, particularly cisplatin‐based chemotherapy, have improved the prognosis of patients with TC, which currently is considered one of the most curable neoplasms.^2^, ^3^, ^4^ According to the International Germ Cell Cancer Collaborative Group (IGCCCG) Update Consortium, up to 90% of patients stratified in the low‐risk group will be cured. Intermediate‐risk patients will have a 5‐year survival rate of approximately 89% and high‐risk patients of nearly 70%.^5^ As a consequence of high survival rates, testicular cancer survivors (TCS) are susceptible to long‐term toxicity of cytotoxic treatment.^6^

Platinum‐based therapies bind to and damage DNA, producing reactive platinum in the serum and healthy tissues, which can be detectable up to 20 years post‐chemotherapy.^7^, ^8^ This persistent hazardous effect could explain the late onset side effects in TCS, which include secondary malignancies, hypogonadism, metabolic syndrome, cardiovascular disease, pulmonary toxicity, neurotoxicity and increased risk of infections.^7^, ^9^, ^10^, ^11^, ^12^, ^13^ Since most patients who are diagnosed with testicular cancer will survive, it is critical to understand mechanisms associated with long‐term toxicity and to exercise caution to minimize avoidable toxicity.^7^, ^14^ Immunosenescence is defined as a decline in the replicative capacity of cells belonging to the immune system with a quantitative and qualitative decrease in effector functions associated with aging.^15^ This phenomenon has been linked to increased susceptibility to infections, decreased response to vaccinations and augmented risk of developing cancer.^16^ An “immunosenescent profile” has been described, which consists of alterations in immune cell subsets. A decrease in naïve CD4^+^ and CD8^+^ cells and increased memory CD4^+^ and CD8^+^ cells have been reported. Both populations also experience a relative increase in terminally differentiated CD57^+^ cells, characterized by a low proliferative capacity and diminished effector response.^17^ Another marker of immunosenescence in numerous immune cell subsets is an increase in the expression of CDKN2A/p16^INK4a^, a cell cycle‐regulating protein.^16^, ^18^ This phenomenon can be mechanistically explained by cumulative DNA damage, which activates the p16INK4a‐encoding *INK4/ARF* (*CDKN2A*) gene on human chromosome 9p21.3, suppressing abnormal proliferating cells.^19^

We previously showed that TCS has increased the expression of *CDKN2A*/*p16* ^*INK4a*^ in peripheral blood lymphocytes compared to healthy controls.^20^ It is unknown if the immune alterations that are present in cancer survivors are associated with the use of chemotherapy or with intrinsic systemic alterations in the immune system related to cancer.^21^ Some patients with TC are treated with orchiectomy alone without being exposed to cytotoxic agents. For this reason, the group of TCS presents a unique opportunity to investigate if different treatment strategies for cancer are associated with the immunosenescent phenotype. This study aimed to determine the association of other treatment modalities with developing an immunosenescent profile in TCS.

## 2 MATERIALS AND METHODS

### 2.1 Patients and healthy controls

A case–control study of TCS compared with age‐matched healthy controls (HC) was performed. TCS were males 18 years or older, with a previously confirmed diagnosis of TC, currently under surveillance for at least 3 months with negative tumor markers and no evidence of disease in a computed tomography scan after the last oncologic treatment. Treatments were orchiectomy only with no cytotoxic therapy provided, chemotherapy with one or more cycles of BEP, or high‐dose chemotherapy with autologous blood stem cell transplantation (BMT). HC were males with no prior history of cancer matched by age (± 12 months). HC had laboratory tests and a clinical evaluation performed by an internal medicine specialist to rule out type 2 diabetes and dyslipidemia. Patients with HIV, Hepatitis B and C, or other chronic diseases were excluded. To minimize bias and adjust for potential confounders, variables such as age, body mass index (BMI), and lifestyle habits (smoking, alcohol consumption, and physical activity) were accounted for in healthy controls to ensure that the observed associations are not due to these confounders.^22^ It has been described that factors like diabetes and hypogonadism could potentially alter *CDKN2A/p16* ^*INK4a*^ expression. To address this, TCS and healthy controls were tested for fasting glucose, and we confirmed that none had diabetes. Likewise, testosterone levels were recorded in average range values, and no patients had clinical criteria for hypogonadism.

This study was approved by the local Institutional Biomedical Research Board (REF. 2499). The study was performed according to the Helsinki Declaration. All subjects were informed about the study's objectives and provided written consent to participate.

### 2.2 Peripheral blood mononuclear cells (PBMC), CD3+ cell purification, RNA extraction/cDNA and *CDKN2A/p16* ^*INK4a*^ analysis

Analysis was performed as described previously.^20^ In brief, a peripheral blood sample (40 mL) was drawn, and Lymphoprep was used to isolate PBMCs by gradient centrifugation (Axis‐Shield PoC AS, Oslo, Norway). Researchers in charge of these assays were blinded to the sample's origin. CD3‐mAb‐coated microbeads (Miltenyi Biotec, Bergisch Gladbach, Germany) were used to purify CD3^+^ cells by positive selection, and flow cytometry was used to confirm purity using an anti‐human CD3‐FITC monoclonal antibody. This procedure routinely yielded preparations with greater than 95% purity.

Total RNA was extracted from CD3^+^ lymphocytes using Trizol (Life Technology, New York, USA) following the manufacturer's instructions. cDNA was synthesized from total RNA using random hexamers as primers and murine leukemia virus reverse transcriptase (MLV‐ RT) (Invitrogen, Carlsbad, CA, USA).

Quantitative expression of *CDKN2A*/*p16* ^*INK4a*^ was determined using the qPCR Taqman assay (TaqMan Universal Master Mix II, with UNG, Applied Biosystems, Foster City, USA) according to the manufacturer's instructions. TaqMan probes were used for *CDKN2A*/*p16* ^*INK4a*^ (exon one alpha‐exon 2; custom order ID: AII1L5T; p16‐FAM‐MGB, Applied Biosystems, Foster City, USA) and GAPDH (GAPDH‐FAM HS 99999901) (Applied Biosystems, Foster City, USA). Samples were run in duplicate in a real‐time polymerase chain reaction (RT‐PCR) instrument, Corbett Research model RG‐6000 (Sydney, Australia), using the program Roto‐gene 6000 version 1.7. The 2^−ΔΔCt^ method was used to assess the relative expression of *CDKN2A*/*p16* ^*INK4a*^ between different study groups.

### 2.3 High sensitivity C‐reactive protein (CRP) analysis

According to the providers instructions, CRP was quantified using an immunoturbidimetry technique with the Beckman Coulter AU System CRP Latex reagent. (Beckman Coulter, Inc., 250 S. Kraemer Blvd. Brea, CA 92821, USA).

### 2.4 Immunophenotyping of leukocyte subpopulations

Peripheral cell immunophenotyping was analyzed as described before.^20^ In brief, EDTA‐treated blood samples were analyzed using an 8‐color flow cytometer (Becton Dickinson Canto II Cytometer) and several combinations of fluorescence‐labeled antibodies from Biolegend Inc. (San Diego, USA). Before lysis (RBC Lysis Buffer, Biolegend Inc., San Diego, USA), 250 μL of blood was incubated with fluorochrome‐conjugated antibodies for 20 min at room temperature and fixed with 3% formaldehyde/PBS. OneFlow TM Setup Beads (BD Biosciences, San Jose, CA, USA) were used to adjust instrument settings. Also, 500,000 events were recorded and analyzed for each sample using FlowJo® v. 10.7.1. (FlowJo, LLC, Oregon, USA). The Flow AI plugin performed an automatic or interactive quality check on the data. An SSC‐A versus FSC‐A plot was used to identify the lymphocyte population and exclude doublets in an FSC‐Height (FSC‐H) by FSC‐Area (FSC‐A) scatter plot.

Leukocyte markers populations were defined as B cells (CD3^−^ CD19^+^), T cells (CD3^+^), CD4 T cells (CD3^+^ CD4^+^), CD8 T cells (CD3^+^ CD8^+^), naïve CD4/CD8 (CD45RA^+^ CCR7^+^), central memory T cells CD4/CD8 (CD45RA^−^ CCR7^+^), effector memory T cells CD4/CD8 (CD45RA^−^ CCR7^−^), terminally differentiated effector memory T cells (TEMRA) (CD57^+^ CCR7^−^), naïve B cells (CD19^+^ CD20^+^ CD27^−^), memory B cells (CD19^+^ CD20^+^ CD27^+^) and plasmoblasts (CD19^+^ CD20^−^ CD27^+^ CD38 high).

### 2.5 Statistical analysis

Continuous data are presented as a median with interquartile ranges (IQR; 25–75). Count and percentage are used to represent categorical values. Every group of TCS was matched 1:1 with healthy controls. For comparisons, paired *t*‐test and Wilcoxon matched‐pairs signed rank tests were used. To adjust for Type I errors, the false discovery rate (FDR) was adjusted using the Benjamini–Hochberg method, with a *q* value set at 0.10. To adjust the comparison of CDKN2A/p16INK4a by age, we performed a linear regression with logarithmic transformation analysis. In order to perform a direct comparison between groups of treatment, we performed a sensitivity intergroup analysis using the Kruskal–Wallis test. GraphPad Prism v.9.0 software was used for these statistical analyses. A *p* ≤ 0.05 was considered statistically significant. To explore the unbiased two‐dimensional clustering of patients, we performed a principal component analysis (PCA) with the standardized values (scaled to a mean = 0 and standard deviation = 1) of the variables that showed differences in any comparison between groups, using BioVinci v3.0.9 (Bioturing, Ca).

## 3 RESULTS

### 3.1 Demographics and clinical characteristics of TCS

We included 65 patients, 19 in the orchiectomy group, 35 in the chemotherapy group, and 11 in the BMT group. The median time on surveillance was 50, 59, and 99 months, respectively. For all groups, non‐seminomatous histology was the most frequent type. The median BMI was in the overweight category across all groups. Table 1 shows the demographic characteristics of patients.

**TABLE 1:** Demographic characteristics of patients.

	Orchiectomy *n* = 19	Chemotherapy *n* = 35	BMT *n* = 11
Median age (min–max)	29 (20–44)	29 (18–45)	25 (18–37)
BMI, kg/m^2^, median (IQR)	25.9 (22.7–31)	25.9 (24.3–29.1)	25.3 (24.2–29.7)
Smoking (%)			
Never	12 (63.1)	20 (57.1)	7 (63.6)
Former	2 (10.5)	10 (28.6)	2 (18.1)
Current	5 (26.3)	5 (14.3)	2 (18.1)
Drinking frequency (%)			
None	10 (52.6)	18 (51.4)	6 (54.5)
Light	4 (21.1)	9 (25.7)	3 (27.3)
Moderate	5 (26.3)	3 (8.6)	2 (18.2)
Heavy	0	2 (5.7)	0
Very heavy	0	3 (8.6)	0
Physical activity (%)			
None	18 (94.7)	31 (88.6)	11 (100)
Light (1.1–2.9 METs)	0	0	0
Moderate (3–5.9 MET)	1 (5.2)	3 (8.6)	0
Vigorous (>6 METs)	0	1 (2.9)	0
Histology			
Seminoma	8 (42.1)	14 (40)	1 (9)
Nonseminoma	11 (57.8)	21 (60)	10 (90.9)
Clinical stage			
I	19 (100)	6 (17.1)	0
II	0	12 (34.2)	0
III	0	17 (48.5)	11 (100)
Chemotherapy regimen			
BEP^a^ 1	0	5 (14.2)	0
BEP ≥3	0	10 (28.5)	3 (27.2)
BEP 4	0	11 (31.4)	0
BEP >4	0	0	0
TIP^b^	0	7 (20)	6 (54.5)
ICE^c^	0	2 (5.7)	0
VIP^d^	0	0	2 (18.1)
Retroperitoneal radiotherapy			
Yes	0	6 (17.1)	3 (27.3)
No	19 (100)	29 (82.9)	8 (72.2)
Retroperitoneal lymph node dissection			
Yes	0	14 (40)	3 (27.3)
No	19 (100)	21 (60)	8 (72.7)
Median testosterone (ng/mL) value (IQR)	3.87 (3.17–4.52)	3.71 (2.87–4.36)	3.36 (2.74–3.64)
Median time on surveillance (months) (min–max)	50 (4–167)	59 (12–213)	99 (40–210)

^a^ Bleomycin, etoposide, and cisplatin.

^b^ Paclitaxel, ifosfamide, and cisplatin.

^c^ Ifosfamide, carboplatin, and etoposide.

^d^ Cisplatin, etoposide, and ifosfamide.

### 3.2 Lymphocyte subpopulations

In the orchiectomy group, data from two patients were excluded from this analysis because of technical issues during acquisition. Among the subsets analyzed, orchiectomy patients only exhibited a lower absolute plasmablasts count (cell/L) compared to HC [orchiectomy 0.12 (0.02–0.22) vs. HC 0.72 (0.32–0.90), *p* = 0.0055].

When compared with healthy controls, patients in the chemotherapy group had a lower absolute count of naïve CD4 T cells (cell/L) [139 (100–274) vs. HC 210 (156–340) *p* = 0.03], and central memory CD8 cells (cell/L) [chemotherapy 16.1 (7.09–164.9) vs. HC 39.8 (20.6–139.6) *p* = 0.02]. Patients in the chemotherapy group had a higher absolute count of effector memory CD4 cells (cells/L) [chemotherapy 115.9 (66.3–295.5) vs. HC 68.6 (33.4–97.3) *p* < 0.0001], effector memory CD8 cells (cell/L) [chemotherapy 97.8 (65–127.8) vs. HC 49.6 (5.6–113) *p* = 0.021], and TEMRA CD8 cells (cell/L) [chemotherapy 28.9 (11.1–46.2) vs. HC 10.8 (0–26.84) *p* = 0.0067]. Within the B cell population, patients in the chemotherapy group had a lower absolute count of memory B cells (cell/L) [chemotherapy 22.8 (14.2–44.8) vs. HC 62.6 (31.2–73.1) *p* = 0.0003] compared to HC.

**FIGURE 1:**
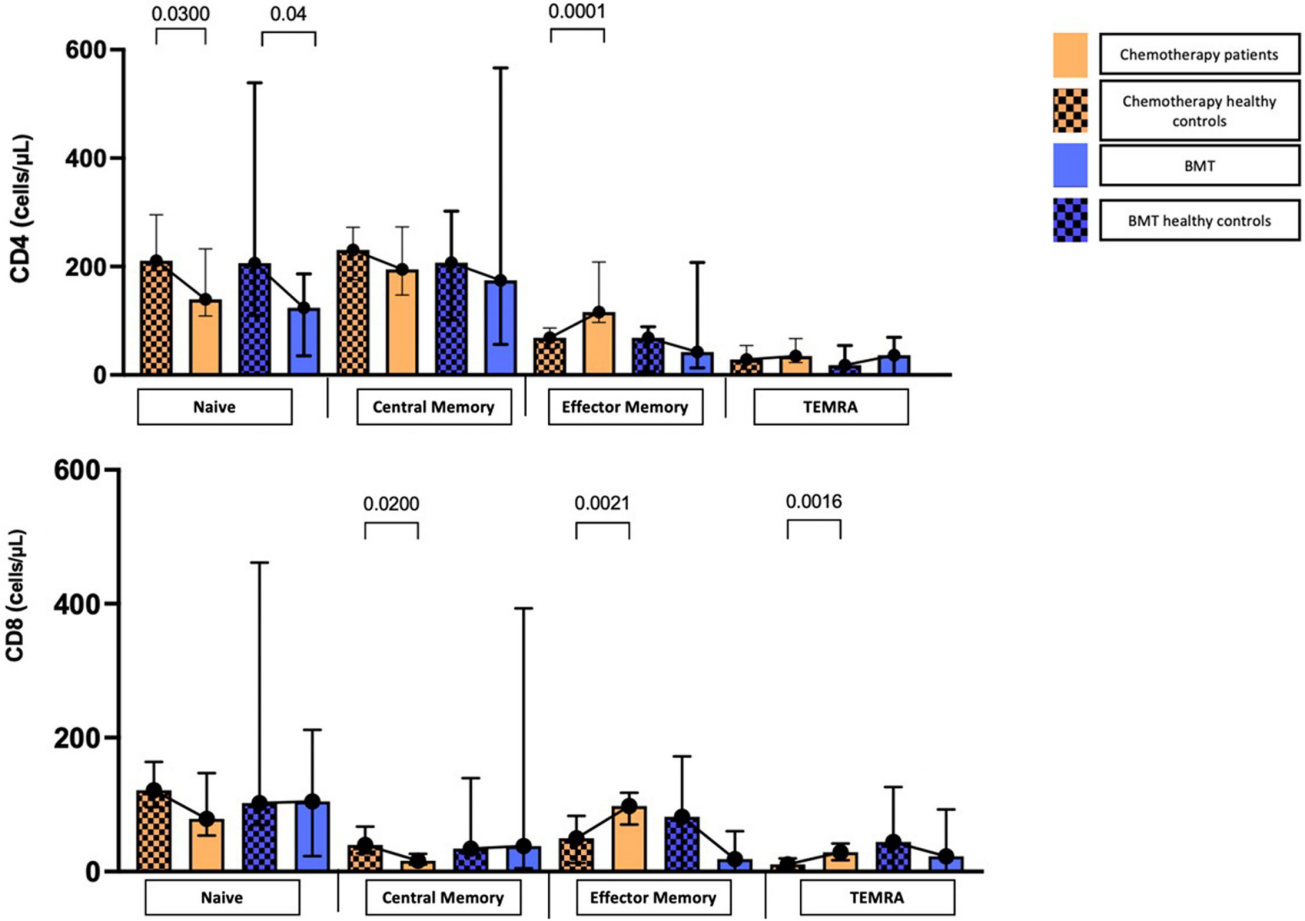
Differences in CD4 and CD8 populations across groups. Determination of CD4^+^ and CD8^+^ cell counts (cells/μL) across groups.

In the BMT group, among the T cell subpopulations, the BMT group had a lower absolute count of naïve CD4 cells (cell/L) [BMT 124.1 (39.6–176) vs. HC 206 (156–369) *p* = 0.04] when compared to HC. Within the B lymphocyte subpopulations, the BMT group had a lower absolute count of plasmablasts (cell/L) [BMT 0.05 (0–0.18) vs. HC 0.37 (0.12–0.90) *p* = 0.0081] compared to HC. Table 2 and Figure 1 depict significant differences across cell populations.

**TABLE 2:** Differences across cell populations between groups.

Cells (%)					Groups (median—IQR)				
	Orchiectomy (*n* = 19)	HC	*p*	Chemotherapy (*n* = 35)	HC	*p*	BMT (*n* = 11)	HC	*p*
Naïve CD4	200 (118–362)	206 (130–336)	0.4307	**139 (100–274)** ^a^	**210 (156–340)**	0.03	**124.1 (39.6–176)** ^a^	**206 (156–369)**	0.0402
CM CD4	240 (208–428)	200 (141–277)	0.0714	230.4 (157.5–282.3)	194.8 (129.3–316.2)	0.8146	174.6 (57.8–427)	207 (149.3–300.9)	0.8311
EM CD4	74.5 (49–91.6)	59.4 (26.4–93.1)	0.4586	**115.9 (66.3–295.5)** ^a^	**68.6 (33.4–97.3)**	<0.0001	42.5 (16.1–176.1)	68.6 (26–80.3)	0.4648
TEMRA CD4	22.8 (4.71–69.8)	28.4 (2.13–147)	0.6777	28.4 (0.7–73.7)	34.9 (16.4–86.2)	0.9549	36.5 (1.2–66.3)	18.2 (0.7–48.7)	0.8265
Naïve CD8	93.4 (51.9–146)	118 (90.8–262)	0.8442	78.9 (41.5–192.6)	121.6 (85.5–205)	0.1442	104.7 (69.8–145.4)	102.2 (69.6–319.7)	0.9658
CM CD8	40.9 (5.58–94)	34.4 (27–176)	0.2247	**16.1 (7.09–164.9)** ^a^	**39.8 (20–6‐139.6)**	0.02	38.1 (6.9–336.9)	34.3 (31.3–67.0)	0.6377
EM CD8	29 (20.5–79)	82 (12.2–128)	0.2435	**97.8 (65–127.8)** ^a^	**49.6 (5.6–113)**	0.021	18.5 (0.9–50.7)	81.9 (11.4–89.7)	0.0663
TEMRA CD8	36 (8.92–74.6)	30.7 (2.21–112)	0.6322	**28.9 (11.1–46.2)** ^a^	**10.8 (0–26.84)**	0.0067	22.6 (0–60.2)	44.3 (0–76.7)	0.5703
Naïve B cells	79.5 (63.5–85.3)	70.6 (63.5–85.3)	0.625	81.5 (74.8–91.9)	71.7 (62.1–78.9)	0.6696	85.5 (77.6–89)	0,6994	0.6994
Memory B cells	31.4 (16.4–61.1)	55.2 (9.51–68.4)	0.6888	**22.8 (14.2–44.8)** ^a^	**62.6 (31.2–73.1)**	0.0003	23.2 (7.84–39.5)	59.8 (23.8–81.1)	0.0652
Plasmablasts	**0.12 (0.02–0.22)** ^a^	**0.72 (0.32–0.90)**	0.0055	0.46 (0.12–1.21)	0.37 (0.03–0.90)	0.8913	**0.05 (0–0.18)** ^a^	**0.37 (0.12–0.90)**	0.0081

*Note:* Bold values intended to highlight significant differences and the *p* value is highlighted in Blue.

Abbreviations: CM, Central Memory; EM, Effector Memory.

^a^ Naïve.

**FIGURE 2:**
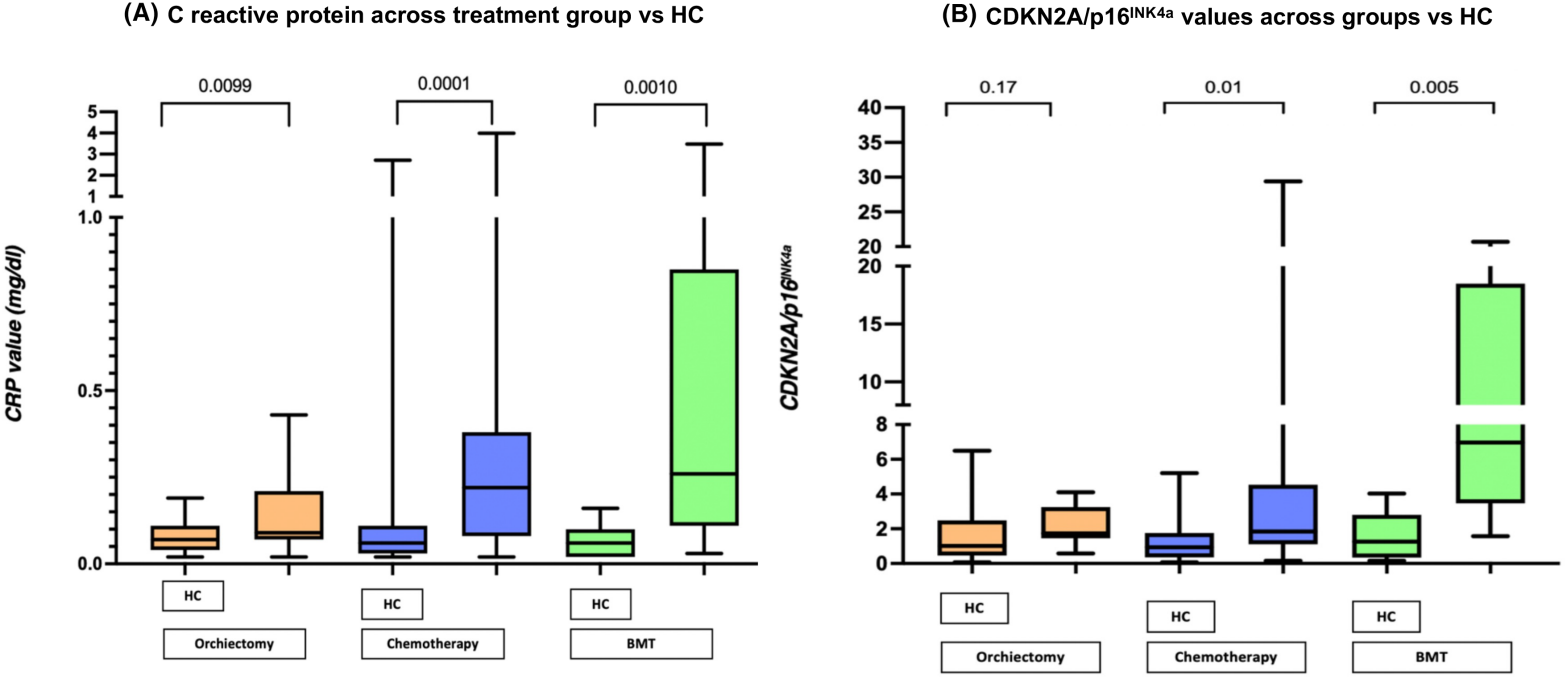
C reactive protein and CDKN2A/*p16* ^*INK4a*^ values in different groups. Determination of C reactive protein levels (mg/dL) across treatment groups.

CRP levels (mg/dL) were higher in all groups of TCS than in HC, although in the orchiectomy group, they only marginally increased. Chemotherapy (median (IQR) 0.22 (0.08–0.38) vs. HC 0.06 (0.03–0.11) *p* < 0.01; BMT group 0.26 (0.11–0.85) vs. HC 0.06 (0.02–0.10) *p* < 0.01; and orchiectomy group 0.09 (0.07–0.21) vs. HC 0.07 (0.03–0.11) *p* < 0.01). These results are shown in Figure 2.

**FIGURE 3:**
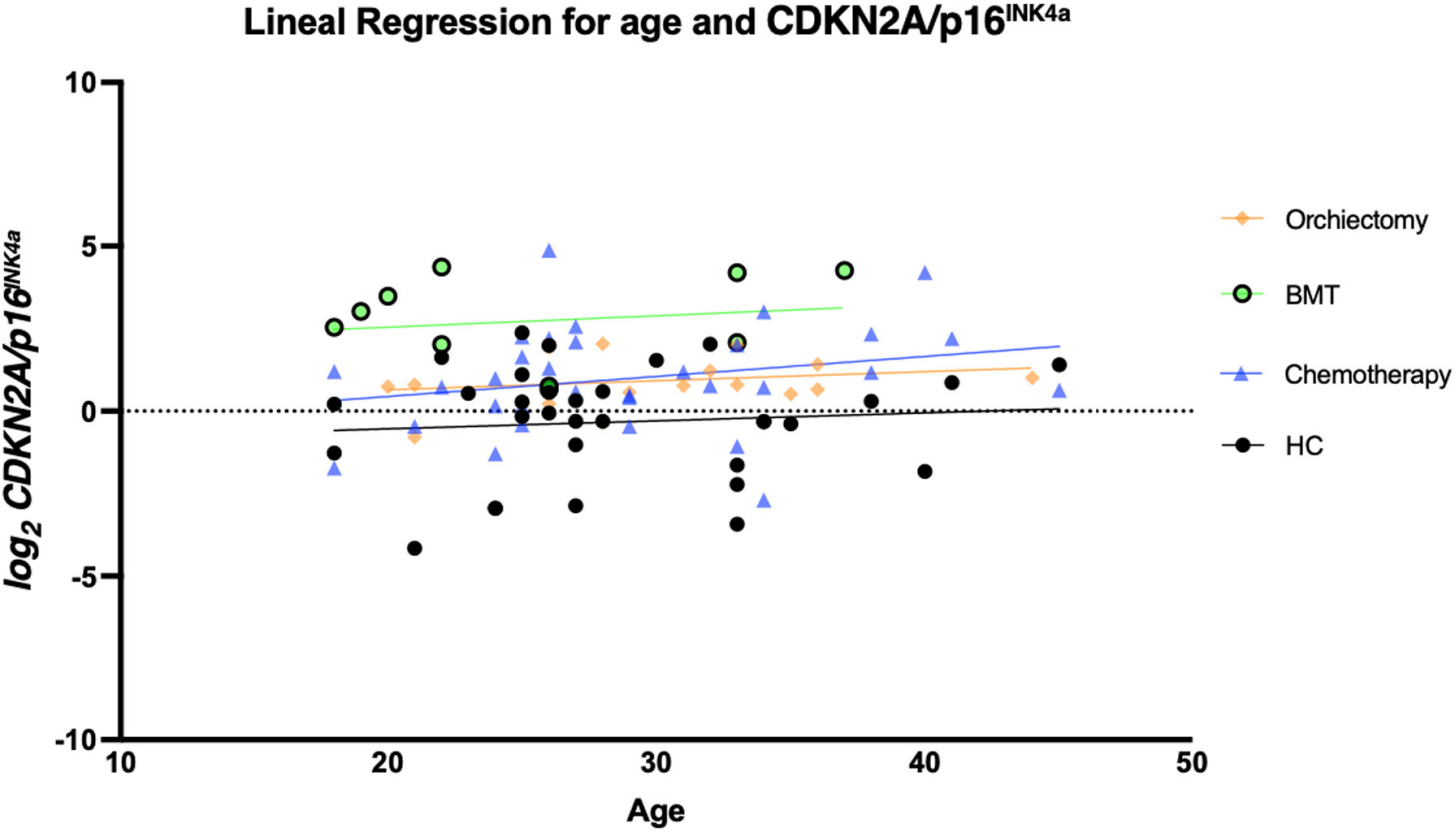
Linear regression for age and CDKN2A/*p16* ^*INK4a*^. Linear regression demonstrating CDKN2A/*p16* ^*INK4a*^ values across groups.

**FIGURE 4:**
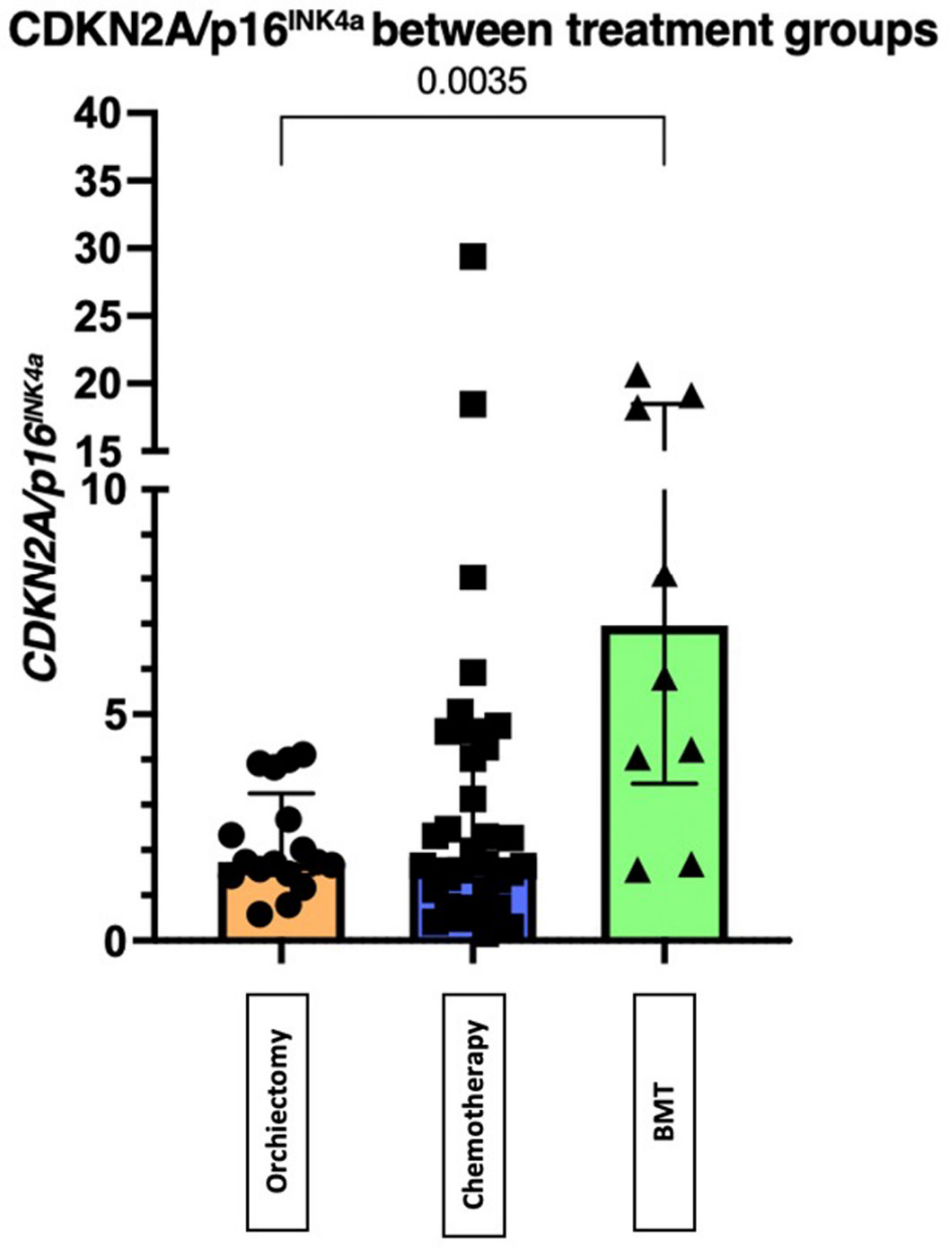
The difference in values of CDKN2A/*p16* ^*INK4a*^ between groups. There is a significant difference in values of CDKN2A/*p16* ^*INK4a*^ between groups.

Due to a lack of cDNA amplification in the RT‐PCR assay, data from three patients in the chemotherapy group, two in the orchiectomy group, and one in the BMT group were excluded from the *CDKN2A/p16* ^*INK4a*^ analysis. Expression of *CDKN2A/p16* ^*INK4a*^ was higher when compared to HC in the chemotherapy group (median (IQR)) [chemotherapy 1.84 (1.11–4.53) vs. HC 0.92 (0.34–1.74) *p* = 0.01] and in the BMT group [BMT 6.96 (3.46–18.48) vs. HC 1.25 (0.33–2.80) *p* < 0.01]. A linear regression analysis of age and *CDKN2A/p16* ^*INK4a*^ expression was performed for all groups, with no statistically significant results. These analyses are shown in Figure 2 and Figure 3. The expression of *CDKN2A/p16* ^*INK4a*^ between treatment groups was significantly different (*p* < 0.01) (Figure 4).

**FIGURE 5:**
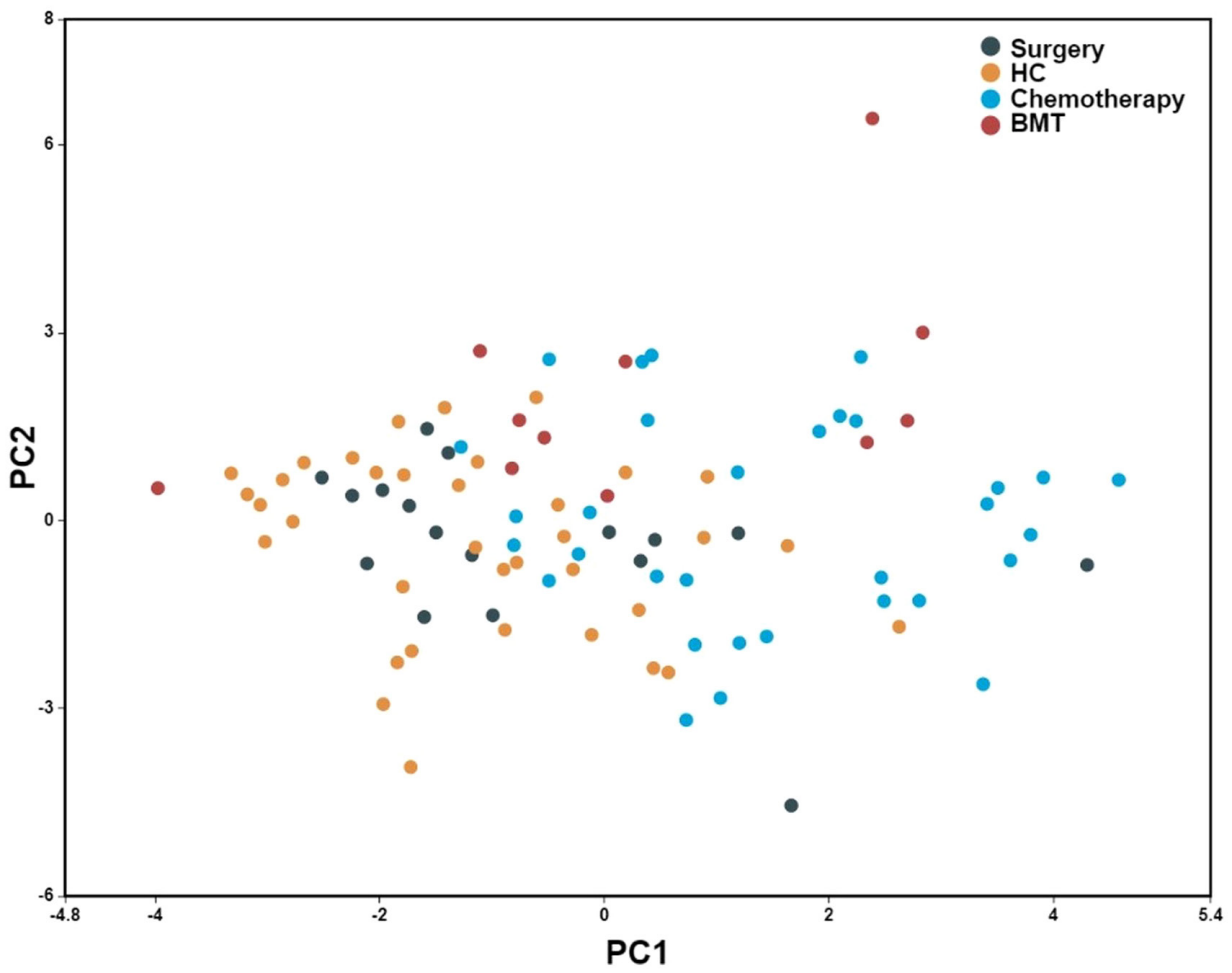
Primary components analysis of cellular populations that showed significant differences between groups. Primary components analysis shows the distribution of cellular populations with significant differences between groups.

To understand the behavior of multidimensional variables across groups, we performed a PCA analysis as described in the methodology section (Figure 5). The first two components explained 39.4% of the observed variance (PC1 explained 22.9% and PC2 16.5% of the variance). In this plot, patients in the orchiectomy group and HC tend to cluster together (moving to the left on the *x*‐axis, PC1), and patients in the chemotherapy group cluster away from those groups (moving to the right on the same axis). The features with higher variance in PC1 were the percentage of naïve CD8 cells, percentage of CD8 memory cells, and percentage of CD8 TEMRA cells.

## 4 DISCUSSION

The results of our study confirm that TCS develop an immunosenescent profile, evidenced by an altered pattern of peripheral lymphocyte subpopulations, and elevated expression of the aging biomarker *CDKN2A/p16* ^*INK4a*^. Our results provide evidence of the immunosenescent phenotype in chemotherapy patients, which is not observed in the orchiectomy group. By evaluating groups with different treatment modalities, we could attribute this alteration to the exposure to cytotoxic chemotherapy. This suggests that immunological aging cannot be solely attributed to testicular cancer itself, but it is a consequence of chemotherapy. The immunosenescent changes observed in testicular cancer survivors treated with chemotherapy might not be unique to this group. Sanoff et al. prospectively evaluated 33 female patients with breast cancer and measured senescent‐related markers, including p16INK4a, before, immediately after, 3 months, and 12 months post‐chemotherapy. They describe a significant increase immediately after chemotherapy that persists through 12 months. Although the authors did not include HC, their results underpin the immunosenescent effect of chemotherapy.^23^ Additional studies are necessary to determine if these changes are also observed in patients receiving similar treatments for other types of cancer.

Patients in both chemotherapy and BMT groups showed significantly increased memory CD4 and CD8 cells and concomitant reduction in naïve CD4 and CD8 cells compared with HC. This profile is concordant with immunosenescence, in which progenitor T cells become less capable of synthesizing new colonies of naïve cells de novo.^24^ Conversely, the same groups showed elevated naïve B cells compared to HC. This might be explained by a lack of T cell‐dependent B cell activation and decreased humoral immunity response.^24^

C‐reactive protein levels were higher in all TCS groups when compared with HC. CRP is considered a surrogate for an inflammatory microenvironment.^25^, ^26^ We did not observe significant differences in any other inflammatory cytokines across groups. It is important to note that even though all groups were significantly different, the relative change observed in the orchiectomy group was smaller compared to the groups that received chemotherapy. “Inflammaging” is a growing concept that refers to the chronic low‐grade inflammation that occurs during aging and its impact on the immune system.^26^ Further research on specific cytokines and their correlation with C‐reactive protein is warranted in the context of the senescence‐associated secretory phenotype (SASP) which can comprise chemokines, extracellular matrix proteases, remodeling factors, bioactive lipids, noncoding nucleotides, and reactive metabolites.^27^ In this regard, removing senescent cells is gaining attention as a potential therapeutic approach to prevent, delay, or reduce various diseases and inflammaging‐related issues. Early successes with senolytics in preclinical studies indicate opportunities to delay multiple chronic conditions and extend a healthy lifespan. Also, suppressing the SASP without eliminating senescent cells is an alternative therapeutic approach for alleviating cellular senescence‐related phenotypes or diseases.^27^

From a mechanical point of view, increased expression of the aging biomarker CDKN2A/*p16* ^*INK4a*^ in chemotherapy and BMT groups can be associated with the cytotoxic effect of chemotherapy, which directly damages DNA and increases the activation of this aging biomarker in healthy peripheral cells.^10^ Our results are consistent with a previous study evaluating patients who underwent chemotherapy and allogeneic stem cell transplantation,^28^ in which this population showed marked increased expression of *CDKN2A*/*p16* ^*INK4a*^. However that study did not include patients with cancer who were not exposed to chemotherapy or healthy controls who were not exposed to cytotoxic therapy. We did not observe an association of *CDKN2A/p16* ^*INK4a*^ with age; this might sound counterintuitive. However, the subjects included in our study are all relatively young, and this association was previously observed in studies that included elderly patients. To confirm that the association was beyond the scope of this study.

It becomes paramount to recognize the long‐term implications of cytotoxic chemotherapy in TCS, as more aggressive treatment could lead to increased susceptibility to complications. The impact of different chemotherapy regimens on cancer patients and whether these alterations remain stable or increase over time is still to be established. Our findings have significant long‐term implications for patient management. Future research focusing on developing specific follow‐up strategies for chemotherapy‐treated testicular cancer survivors is crucial. For example, patients who have received chemotherapy may benefit from regular monitoring of inflammatory biomarkers and adapted vaccination strategies to reduce infection risk. Additionally, interventions to improve immune function, such as regular exercise and proper diet, should be at the forefront during ongoing care.^3^, ^29^ Our results should be interpreted within the broader context of cancer survivorship and long‐term quality of life. It is crucial to recognize that the adverse effects of treatment impact not only physical health but also the psychological and social well‐being of the patient. Future studies should investigate the reversibility of immunosenescent changes and evaluate the effectiveness of specific interventions designed to mitigate the adverse effects of cytotoxic treatment.^30^

Our study has some limitations. There was some heterogeneity in the chemotherapy modality and the dose received by the patients included. There are different doses of BEP, TIP, ICE, and VIP. Despite this heterogeneity, the chemotherapy group exhibits an overall homogeneous behavior, significantly different from matched HC. As mentioned before, understanding how specific regimens or drugs induce immunosenescence will be of interest. The sample size is relatively small, and the number of patients in each group differs, affecting the power to detect differences. However, we conducted two sensitivity analyses, directly comparing different groups of TCS and evaluating the unsupervised clustering of patients according to the variables of interest. Both studies support our conclusions.

## 5 CONCLUSION

TCS exposed to chemotherapy and BMT had an immunosenescence phenotype in peripheral lymphocyte populations compared to HC. Similar patients who received surgical curative treatment alone did not develop an immunosenescent profile. Our results support the attribution of the immunosenescent profile in cancer survivors to cytotoxic chemotherapy. Further research is warranted to understand better the long‐term effect of chemotherapy on the immune system and its impact on the quality of life of TCS.

## AUTHOR CONTRIBUTIONS

**Arturo Carballo Muñoz:** Data curation (equal); formal analysis (equal); investigation (equal); methodology (equal); software (equal); writing – original draft (equal); writing – review and editing (equal). **G. Lima:** Formal analysis (equal); investigation (equal); methodology (equal). **L. Llorente:** Data curation (equal); formal analysis (equal); investigation (equal); writing – original draft (equal). **Yuly Andrea Remolina Bonilla:** Data curation (equal); formal analysis (equal); investigation (equal); writing – review and editing (equal). **Salvador Jaime Casas:** Data curation (equal); formal analysis (equal); investigation (equal); methodology (equal); writing – original draft (equal); writing – review and editing (equal). **Andrea Otamendi Lopez:** Data curation (equal); formal analysis (equal); methodology (equal); writing – original draft (equal). **Ruben Alejandro Ortiz Guerra:** Investigation (equal); methodology (equal). **Hugo E. Velazquez:** Data curation (equal); investigation (equal); writing – original draft (equal). **Y. Atisha‐Fregoso:** Formal analysis (equal); investigation (equal); methodology (equal); writing – review and editing (equal). **M. T. Bourlon:** Conceptualization (lead); formal analysis (equal); investigation (equal); software (equal); supervision (equal); validation (equal); writing – original draft (equal); writing – review and editing (equal).

## FUNDING INFORMATION

This research did not receive funding.

## CONFLICT OF INTEREST STATEMENT

The authors have no conflicts of interest to disclose.

## Data Availability

The data supporting this study's findings are available on request from the corresponding author. The data are not publicly available due to privacy or ethical restrictions.
